# Association of microRNA Polymorphisms with Toxicities Induced by Methotrexate in Children with Acute Lymphoblastic Leukemia

**DOI:** 10.3390/hematolrep15040065

**Published:** 2023-11-20

**Authors:** Vasiliki Karpa, Kallirhoe Kalinderi, Liana Fidani, Athanasios Tragiannidis

**Affiliations:** 1 Laboratory of Medical Biology-Genetics, School of Medicine, Aristotle University of Thessaloniki, 54124 Thessaloniki, Greece; kkalinde@auth.gr (K.K.); sfidani@auth.gr (L.F.); 2 Pediatric & Adolescent Hematology-Oncology Unit, 2nd Pediatric Department, Faculty of Health Sciences, Aristotle University of Thessaloniki, AHEPA Hospital, S. Kiriakidi 1, 54636 Thessaloniki, Greece; atragian@auth.gr

**Keywords:** microRNAs, acute lymphoblastic leukemia, pediatric, methotrexate, toxicity, pharmacogenetics

## Abstract

Methotrexate (MTX), a structurally related substance to folic acid, is an important chemotherapeutic agent used for decades in the treatment of pediatric acute lymphoblastic leukemia (ALL) and other types of cancer as non-Hodgkin lymphomas and osteosarcomas. Despite the successful outcomes observed, the primary drawback is the variability in the pharmacokinetics and pharmacodynamics between patients. The main adverse events related to its use are nephrotoxicity, mucositis, and myelosuppression, especially when used in high doses. The potential adverse reactions and toxicities associated with MTX are a cause for concern and may lead to dose reduction or treatment interruption. Genetic variants in MTX transport genes have been linked to toxicity. Pharmacogenetic studies conducted in the past focused on single nucleotide polymorphisms (SNPs) in the coding and 5′-regulatory regions of genes. Recent studies have demonstrated a significant role of microRNAs (miRNAs) in the transport and metabolism of drugs and in the regulation of target genes. In the last few years, the number of annotated miRNAs has continually risen, in addition to the studies of miRNA polymorphisms and MTX toxicity. Therefore, the objective of the present study is to investigate the role of miRNA variants related to MTX adverse effects.

## 1. Introduction

Pediatric acute lymphoblastic leukemia (ALL) is the most common cancer in children, with approximately 5000 new cases diagnosed annually in Europe alone [1,2,3]. The basic characteristic of ALL is the accumulation of premature lymphoblasts in the bone marrow, which acts as an impediment in blood cell formation [4]. Over the last decades, the survival rates of children with ALL have increased dramatically due to improvements in treatment that essentially consists of a combination of older chemotherapeutics in combination and in high doses with newer targeted therapies and in the amelioration in supportive care [5,6]. ALL treatment in the majority of protocols in the US and Europe consists of three phases: the induction phase, the consolidation phase, and the maintenance phase. After the diagnosis of ALL, the first phase of ALL treatment, the induction phase, entails a multi-drug therapeutic regimen that aims to eliminate the leukemic cells [6]. This phase typically lasts 4 to 6 weeks and is followed by the consolidation phase, which lasts 6 to 9 months and is designed to eradicate any cancer cell that survived from the induction phase. The consolidation phase is followed by the last and longest phase of ALL treatment, the maintenance phase. This stage is designed to diminish the risk of relapse and usually lasts 18 months to 2 years [6,7].

The drugs that are used in the chemotherapy of pediatric ALL are divided into eight different classes, and each of these drugs can be used alone or in combination with other classes in specific parts of the ALL-treatment protocol. Methotrexate (MTX) is an important chemotherapeutic agent amongst the chemotherapeutic drugs that have been used for decades in the treatment of ALL and other types of cancer (e.g., osteosarcoma, colorectal cancer, breast cancer, non-Hodgkin lymphomas) [8,9]. MTX is mostly used during the consolidation and maintenance phase [6,10]. Despite its efficacy, it is known that MTX, which is usually administrated during the consolidation phase, causes a variety of side effects like mucositis, nephrotoxicity, neurotoxicity, hepatotoxicity, bone marrow suppression, etc. [9,10,11].

The pharmacogenomics of MTX has been extensively studied, and common single nucleotide polymorphisms (SNPs) in genes that are implicated in the MTX cellular pathway have been considered to affect the response of the patients to therapy [12]. Recent studies have connected SNPs in miRNAs with the toxicity induced by MTX in children with ALL. In this review, we analyze the association of miRNA polymorphisms with toxicities induced by MTX in children with ALL.

## 2. MTX Mechanism of Action

MTX is a folate antimetabolite that demonstrates exceptional anti-inflammatory, immunosuppressive, and anti-proliferative action [13]. Due to their structural similarity, MTX antagonizes the actions of folic acid, a significant cofactor to a number of enzymes that participate in methionine, thymidine, and purine biosynthesis, as well as in mitochondrial protein translation [2,14,15]. As a result, DNA synthesis and repair are inhibited, the cell cannot divide, and subsequently is led to death [14].

**Figure 1:**
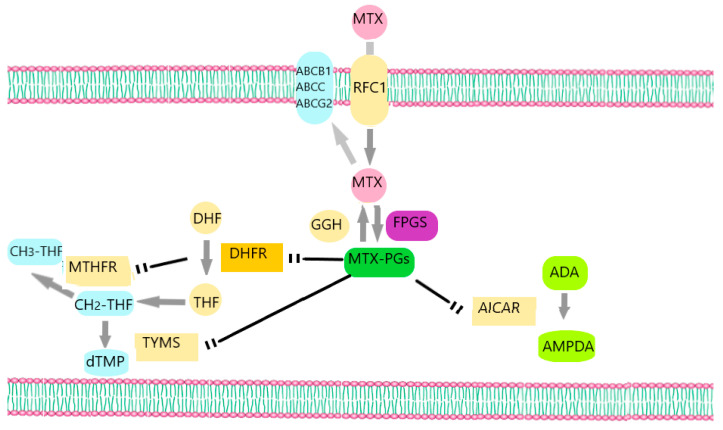
MTX’s mechanism of action. MTX enters the cell via RFC1 and leaves cells through the ATP-binding cassette transporters ABCC, ABCB1, and ABCG2. Intracellularly, MTX is converted into MTX-PGs by the enzyme FPGS, while the enzyme GGH converts MTX-PGs into MTX. MTX-PGs inhibit TYMS, DHFR, AICAR, and MTHFR. ADA: adenine deaminase, AMPDA: Adenosine monophosphate deaminase.

MTX demonstrates a complex mechanism of action due to the large number of its intracellular targets and transporters (Figure 1) [2]. It enters the cell mainly via the most significant transport system for folates that are recognized in mammalian cells, which is the reduced folate carrier (RFC), also known as SLC19A1 [16]. Intracellularly, the enzyme folypolyglutamyl synthetase (FPGS) converts MTX into MTX-polyglutamates (MTX-PGs), while the enzyme γ-glutamyl hydrolase (GGH) catalyzes the conversion of MTX–polyglutamates into MTX and in this way allows the efflux of MTX from the cell [2]. The formation of MTX-PGs inside the cells is important and strongly associated with MTX cytotoxicity [17]. These derivatives, together with MTX, constrain the action of enzymes that are important for many biological processes inside the cell, like DNA replication and repair [13]. One of the target enzymes is dihydrofolate reductase (DHFR), which converts dihydrofolate (DHF) to tetrahydrofolate (THF). THF is a folic acid form that is important for the anabolism of specific amino acids like methionine [4]. Other enzymes inhibited by MTX-PGs are thymidylate synthase (TYMS), which is a key factor in the biosynthesis of pyrimidines; aminoimidazole carboxamide ribonucleotide (AICAR) transformylase, which plays a key role in purine metabolism; and methylenetetrahydrofolate reductase (MTHFR), an enzyme involved in amino acid metabolism [4,18].

## 3. MTX in ALL Treatment—Adverse Effects

MTX, in combination with other drugs such as corticosteroids, vincristine, and L-asparaginase, are the cornerstone of ALL treatment and have undeniably led to the improvement in the survival rates of patients [19]. MTX can be administrated orally, intramuscularly, intravenously, or intrathecally at doses that depend on the protocol followed. Usually, doses that are <500 mg/m^2^ are considered to be low (LD-MTX), doses of 500–1000 mg/m^2^ are classified as intermediate, while doses ≥1000 mg/m^2^ are defined as high dose MTX (HD-MTX) [20]. In ALL treatment, MTX can be administrated at doses intravenously as high as 33,000 mg/m^2^/24 h [19]. In the recent BFM protocols, doses vary from 2000 to 5000 mg/m^2^/24 h every 14 days according to the patient’s immunophenotype and risk of disease (https://www.bialaczka.org/wp-content/uploads/2016/10/ALLIC_BFM_2009.pdf, accessed on 9 September 2023). Adverse effects of MTX administration are usually related to high doses of MTX, but toxicities are documented even with the administration of low to intermediate dose MTX [20]. Low-dose MTX regimens can cause moderate bone marrow suppression, pulmonary toxicity (0.5% of the patients per year), and liver damage, while the administration of high-dose MTX is related to emesis (in 10–30% of the patients), severe bone marrow suppression, renal toxicity (acute kidney injury in 2–12% of the patients), neurotoxicity (in up to 15% of HD-MTX courses), liver damage (reversible chemical hepatitis in up to 60% of HD-MTX courses; hyperbilirubinemia in 25% of HD-MTX courses,), and mucositis [9,21,22]. One-third of the patients that receive HD-MTX will exhibit side effects, and this situation can be so severe that it will lead to death in 1–3% of them [19].

The absorption, distribution, metabolism, and excretion of MTX determine the level of post-treatment support and follow-up. Plasma concentrations of MTX after a fixed-dose, fixed-time HD-MTX infusion can differ significantly from patient to patient and even within a single patient on multiple cycles of therapy. Because MTX is mainly eradicated from the body by the kidneys, renal function should be evaluated before, during, and after each HD-MTX cycle [22].

It is crucial to detect any abnormality early enough so that an immediate intervention can be made and, in this way, prevent any damage to the kidneys. Urine alkalinization and effective hydration aim to increase MTX excretion from the body and to protect kidney function [21]. Another measure for the prevention of MTX toxicity is the administration of leucovorin. Leucovorin is a folate analog that has been used for more than 30 years to counterbalance the toxicity of treatment with HD-MTX [22]. The purpose of leucovorin administration during HD-MTX therapy is to “rescue” normal cells from the action of MTX and to reduce the side effects of MTX. That is the reason why it is frequently known as leucovorin rescue. The dose and frequency of leucovorin administration are defined and can be altered in therapeutic protocol with HD-MTX [22]. Leucovorin can interfere with the therapeutic action of MTX, which is why it is usually administrated 24 h after the administration of MTX [23].

Other complementary individualized care strategies can be followed when needed. In patients, for example, that have already been diagnosed with baseline kidney disease and renal impairment or manifested nephrotoxicity as a side effect after administration of previous HD-MTX, the dose of MTX that is to be administrated during therapy should be reduced. In addition, in order to ensure that there is not delayed MTX clearance, the monitoring of MTX levels in serum can be measured earlier than usual, for example, in the sixth hour after the infusion [22]. Equally important is the avoidance of using other drugs during treatment with HD-MTX, which can potentially make MTX clearance less efficient and consequently act as nephrotoxins. This category of drugs includes proton pump inhibitors, non-steroidal anti-inflammatory drugs, penicillin and its derivatives, salicylates, amphotericin, aminoglycosides, levetiracetam, chloral hydrate, and radiographic contrast dyes. The main nephrotoxic drugs that are known to cause a decrease in MTX clearance and are usually used as supportive care and antiinfective prophylaxis and treatment are antibiotics as aminoglycosides and antifungals as liposomal amphotericin B.

Briefly, although MTX represents one of the main chemotherapeutics of pediatric ALL treatment and is the cornerstone for central nervous system prophylaxis via leukemic blasts, its toxicity is a major obstacle for a successful therapy and should be managed appropriately or, if possible, predicted before the manifestation of its side effects. A few studies, mainly in patients with leukemia, broached the possibility of individualized MTX treatment according to the patient’s genetic makeup. A lot of research has focused over the last few years on the association of genetic variants with MTX toxicity [3]. The results of various studies have indicated that genetic variants play an important role in disease prediction as well as gene-to-gene interactions, which may play an even more significant role in disease prediction than individual genes [24].

## 4. Pharmacogenetics of MTX

Pharmacogenetics refers to the study of drug response variability due to inheritance. The most common type of genetic variation is SNP, where a single base has been substituted or removed or an additional base has been added. SNPs are estimated to occur every 500–1000 bases in the human genome and can be used to explain variations in disease susceptibility across a population. SNPs may be found in the coding and non-coding regions of a gene or even in the region between two genes, with different impacts in each case. SNPs in the coding region may result in amino acid changes (non-synonymous polymorphisms). SNPs in non-coding areas may still have an effect on gene transcription, gene splicing, or RNA stability. Thanks to rapid advances in high throughput genotyping, large numbers of SNPs across the human genome can now be screened and analyzed for their potential to affect drug response [24].

There is a well-established relationship between MTX toxicity and polymorphisms in genes that participate in the pathways by which MTX is absorbed, metabolized, and excreted out of the body. In addition, several studies reported an association between SNPs in genes that are implicated in MTX’s mechanism of action and the drug’s efficacy and toxicity [8]. In the era of precision medicine, knowing an individual’s genotype can be of great importance in order to administer the right dose of MTX with fewer adverse effects and maximum efficacy. Among the genes that are well studied are the MTHFR gene, the SLCO1B1 gene, the TYMS gene, and the DHFR gene, as well as the SLC19A1, which are analyzed below.

### 4.1. The Methylenetetrahydrofolate Reductase (MTHFR) Gene

MTX is indirectly inhibited by the MTHFR enzyme. The byproduct of this process is 5- methyl-tetrahydrofolic acid or 5-CH3-THF. This byproduct takes part in the remethylation of toxic homocysteine to methionine and is, in general, a methyl group donor to several molecules [4,25,26]. Many SNPs in the MTHFR gene have been identified, but the most common of them are C677T (rs1801133) and A1298C (rs1801133) [27]. In C677T polymorphism, a nucleotide with cytosine (C) is substituted by a nucleotide with thymine (T), and as a result, instead of an alanine in the same position (at codon 222) in the enzyme, there is a valine. Similarly, in the A1298 polymorphism, a nucleotide with adenine (A) is substituted by a nucleotide with cytosine (C), and consequently, instead of glutamic acid at codon 429, there is alanine. These polymorphisms have been well studied for their association with the therapeutic outcome in patients with ALL when treated with MTX, but the results are contradictory [4,28]. The majority of reports that included adult patients with ALL indicated that the MTHFR genotype rs1801133 T was associated with increased toxicity and reduced efficacy [4,25]. On the other hand, when it comes to pediatric ALL patients, the results of the studies are in contradiction with the ones that include adult patients. In children with ALL treated with MTX, no association between rs1801133 T and drug toxicity was reported, but a decreased risk for hematological toxicity was associated with the 1298CC genotype, as it is shown in a few studies [4,25,29]. In fact, the vast majority of published studies do not indicate a relationship between polymorphism in MTHFR and toxicity in pediatric ALL [30]. Studies that do find an association between this gene and MTX toxicity usually conflict with one another [28,29,30,31]. Thus, the inconsistency of the results reported to date makes it difficult to draw any definitive conclusions on the association of MTHFR variants with drug toxicity and efficacy.

### 4.2. The Organic Anion Transporter Family Member 1B1 (SLCO1B1) Gene

Organic anion-transporting polypeptide OATP1B1, also known as SLCO1B1, is a sodium-independent uptake membrane transporter and a member of the OATP family. It is primarily expressed in the human hepatocytes (in the basolateral membrane) but can also be found in the small intestine (enterocytes) [32]. SLCO1B1 is a polymorphic transporter that takes part in the translocation of a wide variety of both endogenous and exogenous compounds. The endogenous substrates of SLCO1B1 include bilirubin, bile acids, and the thyroid hormones thyroxine and triiodothyronine, while the exogenous compounds include basically drugs such as 3-hydroxy-3-methylglutaryl-co-enzymeA (HMG CoA)-reductase inhibitors, angiotensin-converting enzyme (ACE) inhibitors, and MTX [32,33].

Functional and ethnically dependent polymorphisms of SLCO1B1 have been characterized and identified. Clinical studies confirm that SLCO1B1 variants are associated with changes in substrate drug pharmacokinetics, response to treatment, and risk of drug-induced toxicities [34]. In particular, in a genome-wide association study that included 434 pediatric ALL patients, the T allele in rs11045879 polymorphism and G allele in rs4149081 polymorphism were each associated with gastrointestinal toxicity during the consolidation and continuation phases of ALL treatment protocol [4,35]. In another study conducted in 115 Spanish pediatric ALL patients treated with MTX, both rs4149081 AA and rs11045879 CC genotypes were associated with increased MTX plasma levels [36]. These results have been replicated in numerous studies, indicating that these SLCO1A1 variants play an important role in predicting MTX elimination and toxicity [4,28]. In addition, four large studies conducted in pediatric ALL patients showed that the CC rs4149056 genotype is associated with decreased MTX clearance [35,37,38]. Other SNPs in SlCO1B1(rs2306283, rs11045872) were studied for their association with the drug’s clearance, but more research needs to be conducted in order to reach definite conclusions [25]. All these findings indicate the significance of SLCO1B1 variants as a reliable genetic marker for MTX toxicity and MTX clearance [19].

### 4.3. The Thymidylate Synthetase (TYMS) Gene

Thymidylate synthetase (TYMS) is a folate pathway enzyme and is responsible for the formation of deoxythymidylate monophosphate (dTMP) from deoxyuridylatemonophosphate. It plays an important role in DNA biosynthesis, DNA replication, and cell proliferation. TYMS is inhibited by MTX and MTX-PGs, which consequently results in the depletion of deoxythymidine triphosphate and the suspension of DNA replication [4].

The possibility of TYMS polymorphisms to be linked to the efficacy and toxicity of MTX has been investigated in several studies. Amongst the most common polymorphisms in the gene are the 2R/3R polymorphism (rs34743033) and the 6 bp insertion/deletion (I/D) polymorphism (rs16430). The first one is a double (2R) or triple (3R) 28 base pair repeat in the 5′ untranslated region, while the second one is a 6 base pair sequence deletion in the 3′ untranslated region of the gene [39]. These polymorphisms have been associated with differences in TYMS expression, as well as treatment response and MTX toxicity in several studies [40,41,42,43,44]. Other studies connected TYM polymorphisms with other complications during therapy. For example, a study that included 127 Lebanese children with ALL showed that there was a need to decrease weekly MTX doses in TYMS 28-bp tandem repeat carriers [45]. Another study reported an increased risk of hepatotoxicity in 2R/3R and 3R/3R carriers in rs34743033 polymorphism, as well as more numerous vomiting episodes in 3R/3R carriers [46]. When it comes to the TYMS 6bp deletion, a recent study conducted in a group of 148 pediatric patients concluded that patients with a homozygous TYMS 6bp deletion were more susceptible to gastrointestinal toxicity [47]. Given all that, TYMS gene polymorphisms seem to be a promising marker for MTX toxicity and efficacy.

### 4.4. The Dihydrofolate Reductase (DHFR) Gene

The dihydrofolate reductase (DHFR) enzyme is encoded from the homonym gene and is an enzyme that catalyzes the reaction of tetrahydrofolate (THF) production from dihydrofolate (DHF). MTX and its PGs act as competitive inhibitors of the DHFR enzyme, causing a decrease in the ability of THF production from DHF. The absence of THF associated with MTX therapy interferes with many steps of DNA synthesis and DNA methylation, resulting in one of MTX’s therapeutic effects, namely cell death [47].

Altered levels of DHFR have been observed in patients with relapsed disease and in cells with an MTX-resistant phenotype in both experimental and clinical trials. This suggests that DHFR plays an important role in the development of resistance against MTX [48]. Alterations in the expression of DHFR, and thus in the susceptibility to MTX, may also be caused by polymorphisms in the homonym gene, especially those found in its regulatory regions. A number of studies have demonstrated that there is an association between specific polymorphisms in the DHFR gene (A-317G, C829T, C-1610G/T, and C-680A) and treatment outcome [49,50,51]. However, these studies found no association between these polymorphisms and drug toxicity.

### 4.5. Reduced Folate Carrier 1 (RFC 1) Gene

Reduced Folate Carrier 1 (RFC1), also known as the solute carrier family 19 member 1 (SLC19A1), is a membrane protein that helps regulate the transport of folates. It also plays an important role in the uptake of antifolate chemotherapy drugs like MTX into cells [52]. One of the most common SNPs in the gene is the RFC1 80G > A (r1051266) polymorphism in which guanine is substituted for adenine (at nucleotide 80), and arginine is substituted for histidine (at protein residue 27) in the protein. This results in a decrease in the transport of antifolate chemotherapy agents [52,53,54,55]. A few studies have shown a relationship between the RFC1 (80G > A) polymorphism and a risk for pediatric ALL [52,53,54,55]. However, in a metanalysis that included data from 10 case–control studies, no relationship was found between G80A in RFC1 and the risk of ALL, as well as there was no relationship in the sub-analysis by ethnicity [56].

Several other studies have investigated the relationship between this polymorphism and risk for ALL relapse, MTX toxicity, and survival rates, and the results, again, are controversial. In a study looking at the effects of RFC1 polymorphisms (SNPs) and haplotypes on toxicities induced by HD-MTX in 88 pediatric patients with ALL, the authors found that the TT genotype in rs2838958 polymorphism was associated with a higher risk of mucositis in the patients compared to carriers who had at least one C allele in their genotype. Moreover, they found that haplotype 4 (TGTTCCG) significantly reduced the risk of adverse reactions during the treatment with HD-MTX [57]. In another study conducted in 182 children with ALL treated with HD-MTX, bone marrow toxicity was observed at a higher frequency in RFC AA variant patients compared to GA/GG variant patients, while liver toxicity was observed in RFC GG variant patients [58]. In addition, a study performed by Kishi et al. found that the RFC 80A allele is strongly associated with gastrointestinal toxicity [59]. Nevertheless, two studies, one by He et al. and the other by Chiusolo et al., concluded that there is no association between the RFC1 80G > A polymorphism and MTX toxicity [60,61].

To sum up, in recent years, there have been numerous studies on the association of genetic variation with treatment-related toxicities in ALL. SNPs in many genes that are involved in MTX’s mechanism of action have been studied thoroughly for their association with MTX toxicity and efficacy. Polymorphisms in the MTHFR gene have been investigated for their relationship with the therapeutic outcome in patients with ALL, but the results are contradictory. Clinical studies confirm that SLCOB1 polymorphisms, as well as TYMs variants, are related to treatment outcome and toxicity, indicating that these gene variants can serve as reliable genetic markers for MTX toxicity and clearance. DHFR SNPs were associated only with treatment outcome, but no correlation was found between these polymorphisms and the drug’s toxicity. When it comes to RFC1 gene polymorphisms, the results of the studies investigating the relationship between RFC1 variants and MTX toxicity, risk for ALL relapse, and survival rates are controversial.

The vast majority of these studies focus on coding regions (which account for approximately 1.5%). To date, it has become clear that non-coding protein regions play an important regulatory role. For instance, some genes, including transporter genes like SLC19A1, are regulated post-transcriptionally by miRNAs, and miRNA variants may alter miRNA levels or functions. Over the last few years, there has been a significant increase in the number of miRNAs annotated, and the research about the association of miRNAs and MTX efficacy and toxicity has bloomed [3]. This is a novel field of interest that suggests that miRNAs can be a promising predicting tool for MTX adverse events and effectiveness.

## 5. miRNAs Biology and Role in Cancer

miRNAs, as indicated by their nomenclature, are a category of small, non-coding, single-stranded RNAs with a length of 18–25 nucleotides [62]. The process of miRNA biogenesis includes multiple stages [63]. The first step involves the transcription of miRNA genes to long primary transcripts that are called pri-miRNAs. Then, the pri-miRNAs are converted to pre-miRNAs. These pre-miRNAs are stem-loop precursors of 70–90 nt that are exported to the cytoplasm of the cell. In the cytoplasm, DICER (ribonuclease involved in the miRNA processing) cleaves the pre-miRNAs to produce mature miRNAs that consist of 22 nt [64,65].

Approximately 2600 miRNA genes are encoded by the human genome [66] and regulate one-third of the human genome [67]. Their role is essential in the human body as they regulate gene expression, transmit information from one cell to another (intercellular signaling), and participate in many biological functions [68,69]. miRNAs moderate gene expression at the level that follows DNA transcription by attaching to the 3′ untranslated area of specific mRNAs. This results in the degradation of the mRNA or inhibition of the mRNA translation.

More evidence has shown that the presence of SNPs in pre- and mature miRNAs can potentially affect a variety of biological pathways by affecting target selection or mature miRNA abundances. Because miRNAs play such a critical role in regulating gene expression, they must be tightly regulated. As a result, poor miRNA expression regulation leads to highly abnormal gene expression and human diseases (e.g., cardiological and auto-immune diseases) [48,70].

Alterations in miRNA expression have also been linked to cancer progression, where global reduction in miRNA expression is typically observed [71,72,73,74,75]. For instance, a number of studies have suggested that miRNAs play a role in the transformation of malignant T cells in ALL, including the discovery of an oncogenic miRNA network (miR-19b, mir-20a, miR-26a, miR-92, and miR-223) and an inhibitory miRNA network (miR-150, miR-155, miR-200, and miR-193b-3p) associated with T-ALL disease biology [76]. Because of their overexpression or downregulation in cancer, miRNAs have great potential to be biomarkers for early diagnosis of malignancies because they can be identified and extracted from the blood (in total, plasma, or serum) [77]. Lawrie et al., in their study published in 2008, identified for the first time that miR-21 showed an abnormal expression in patients with large B-cell lymphoma, suggesting that it could be a potential biomarker for the early detection of this malignancy. Following this, more research has identified a growing number of miRNAs circulating in the blood as potential leukemia biomarkers [78]. In childhood ALL, there is a significant difference in expression levels between miR-100 and miR-99a, with very low expression levels observed in ALL patients compared to acute myeloid leukemia (AML) patients or healthy bone marrow donors. Furthermore, the expression levels of two other tumor suppressive miRNAs (miR-326 snf miR200c) appear to significantly decrease in bone marrow cells of pediatric ALL patients. Therefore, these miRNAs may play an important role in leukemogenesis and may act as a potential reliable biomarker for pediatric ALL [79].

miRNAs could also be used for the diagnosis and classification in pediatric ALL. Specifically, three miRNAs (miR-128a and miR-128b) were significantly overexpressed in ALL compared with AML, while one (miR-223) was significantly downregulated. The results demonstrate that ALL can be distinguished from AML with a 95% confidence interval based on the differential expression pattern of all four miRNAs [80]. In addition, in childhood ALL, there is a significant difference in the level of expression of two miRNAs (miR-100 and miR-99a) compared to healthy individuals. Patients with ALL showed significantly lower levels of expression than those with AML or healthy bone marrow donors. Furthermore, it appears that the levels of two other tumor suppressors (miR-326 and miR-200c) are significantly downregulated in pediatric ALL bone marrow mononuclear cell samples at diagnosis, while the sensitivity and specificity of both miRNAs in ROC (area under the curve) analysis indicated that they might be potential reliable biomarkers for pediatric ALL [80].

An analysis of miR-203 and miR-125b expression levels in peripheral blood that was isolated from 43 newly diagnosed children with ALL showed a 33-fold increase in miR-125b expression in ALL cases compared to the healthy control group and a 31-fold increase in miR-203 expression in the control group compared to ALL cases. While miRNA-203 sensitivity was higher than miRNA-125b sensitivity, their combination showed absolute sensitivity, indicating that preclinical studies focused on miRNAs for diagnosis of ALL need to be strongly supported [80].

Currently, pediatric ALL is stratified based on various biological and clinical criteria, with patients receiving risk-adjusted therapy. While current strategies provide high cure rates, there are many patients with ALL who relapse (16.7–24.5%) [81]. The traditional use of gene expression profiling (GEP) and mRNA signature in the clinical environment has drawbacks, and only modest success has been observed. As miRNA profiling tends to be restricted to a few genes, it may be able to provide fewer and more powerful signatures that have the same high prognostic power.

The downregulation of miR-128b and the overexpression of miR-16 and miR-223 have been correlated with good prognosis and longer disease-free and overall survival. On the other hand, an increase in expression of miR-24 and miR-155 and the downregulation of miR-335, miR-326, miR27a, and miR-125b can lead to increased risk of relapse, poor response to therapy, increased incidence of drug resistance, and is correlated with poor disease prognosis (Table 1) [80].

## 6. miRNAs, Drug Response, and Toxicity

A few years ago, pharmacogenetic studies focused on SNPs in the coding region of genes, as well as in the 5′-regulated region of genes. New evidence shows that SNPs in miRNAs that can bind to the 3′UTR of genes can affect the risk of disease, as well as drug responses. Recent studies proved that miRNAs play an important role in drug transport and metabolism, as well as in the regulation of target genes. As a result, it was suggested that miRNAs can play a significant role in treatment response. For instance, miRNAs have been shown to interact with a variety of drugs, including vincristine, daunorubicin, and glucocorticoids. To be more specific, studies conducted in children with B-ALL showed that an increase in the expression of miR-125b, miR99a, and miR-100 was associated with resistance to vincristine and daunorubicin, while a decrease in the expression of miR-708 was related with resistance to glucocorticoids [82,83]. These data suggest that alterations in miRNA expression or function could influence response to treatment [28].

To date, several studies have investigated the association of miRNA polymorphisms with toxicities induced by MTX in children with ALL. Hepatotoxicity is one of the MTX-induced adverse effects and is developed in almost two-thirds of ALL pediatric patients during therapy and specifically in the course of the consolidation therapy. A large number of genes involved in the normal liver-dependent signaling pathways are highly regulated by the miRNAs and have been shown to be susceptible to modulation via miRNA gene sequence variations. For example, there is a strong association between high transaminase toxicities during the consolidation phase of MTX treatment (but not the induction phase) and the presence of the rs264881 polymorphism that modulates stability in the pre-miRNA of miR-1208. As expected, miR1208 targets include genes involved in the pharmacodynamics and pharmacokinetics of MTX; one of the primary targets of MTX is, as mentioned before, DHFR. It is possible that increased miR1208 expression could potentially attenuate the adverse effects of MTX that arise from DHFR inhibition [80].

Many other nucleotide polymorphisms in miRNAs have been demonstrated to be useful in predicting ΜΤΧ-related toxicity. Multiple studies have demonstrated a correlation between rs2114358 polymorphism in miR-1206 and oral mucositis caused by MTX [84]. This toxicity is characterized by the disruption of the mucosa, leading to extensive ulceration in the oral cavity and gastrointestinal tract. As a result, children experience abdominal pain, vomiting, and diarrhea, resulting in weight loss, nutritional deficiencies, and a heightened risk of infection. Camino et al., in 2018, investigated the involvement of 160 miRNA SNPs in mucositis development in 179 Spanish children diagnosed with B-ALL who were treated with the Spanish standard LAL-SHOP protocols. The results showed that rs4674470 in miR-4268 was associated with a reduced risk of mucositis toxicity, while rs8667 in miR-4751 was associated with an increased risk of developing diarrhea. In addition, rs12402181 in miR-3117 was associated with a decrease in the risk of developing vomiting [85]. Actually, this is a vital finding because oral mucositis is a common side effect present in 20% of pediatric ALL patients treated with MTX [28,86].

A new study (2023) conducted by Zhan et al. evaluated the relationship between 15 SNPs in miRNAs and hematological toxicities induced by HD-MTX in 181 Chinese pediatric patients with ALL [65]. The results indicate that rs2114358 G > A polymorphism in pre-hsa-miR-1206 was associated with grade 3/4 leukopenia while rs56103835 T  >  C polymorphism in pre-hsa-mir-323b was associated with grade 3/4 anemia (with the use of multiple logistic regression). Furthermore, with the help of bioinformatics, they concluded that rs2114358 G > A polymorphism will affect the secondary structure of pre-miR-1206 and rs56103835 T > C polymorphism will, respectively, affect the secondary structure of pre-miR-323b. This phenomenon will possibly interfere with the expression of mature miRNAs and, consequently, with the expression of the miRNAs’ target genes. These results show clearly that rs2114358 G > A and rs56103835 T > C polymorphisms in miR-1206 and miR-323b, respectively, are candidate clinical biomarkers for the prediction of grade 3/4 hematological toxicity in pediatric patients with ALL [65].

Another study published in 2023 and conducted by da Silva Menezes et al. examined the influence of 25 SNPs on the miRNA genes and proteins encoded by the miRNA synthesis complex in 77 Brazilian B cell ALL patients receiving treatment (MTX, 6-MP, or both) [87]. The concluding data indicate that rs229283 polymorphism in miR-149, rs2043556 polymorphism in miR-605, rs10505168 polymorphism in miR-2053, and rs2505901 polymorphism in miR-938 have a big impact on neurological toxicity during ALL treatment while the rs2505901 variant of the miR-938 gene was significantly associated with protection from gastrointestinal and neurological toxicity. Furthermore, rs56103835 in miR-323B and rs639174 in DROSHA (encodes a ribonuclease for the conversion of pri-miRNAs to pre-miRNAs) were strongly associated with the risk of developing gastrointestinal toxicities in patients [87]. The connection between the rs639174 polymorphism in the DROSHA miRNA processing gene was examined in a previously published study with the same results, indicating that it could be a good indicator of MTX-induced gastrointestinal toxicity [87,88]. When it comes to hematological toxicity, the results revealed substantial data on the risk of hematological toxicity associated with the variants rs12904 in miR-200C, rs3746444 miR-499A, and rs10739971 miR-LET7A1 associated with ALL treatment. Specifically, rs12904 in miR-200C was found to be 74% less likely to result in hematological toxicity. In addition, rs3746444 polymorphism in miR-499A was found to be 77% less likely to cause toxic hematological events during treatment. Last but not least, the presence of the rs10739971 variant of the miR-LET7A1 gene was related to an 82% reduction in the risk of developing hematological toxicities (Table 2) [87].

miRNA genetic polymorphisms have been shown to alter the plasma levels of MTX by modulating the carriers of MTX in all pediatric patients with ALL. An effective indicator of MTX clearance and exposure during the treatment period is plasma MTX concentration. Thus, genetic variations that influence plasma levels of MTX are important to determine the appropriate dose of MTX, as well as the duration of leucovorin rescue, in order to have an effective therapy with minimum drug toxicity effects. Iparraguirre et al. found that three miRNA polymorphisms, rs56292801 in miR-5189, rs4909237 in miR-595, and rs78790512 in miR-6083, are related to plasma levels of MTX. For miR-5189, the presence of the AA/AG genotype was associated with a significantly reduced risk of MTX plasma levels (0.4-fold), while for miR-595, the rs4909237 TT genotype was linked to an increased risk of 5.7 times higher plasma MTX levels. When it comes to miR-6083, the rs78790512 AA genotype was never present in patients with high levels of MTX in their blood, suggesting that this specific genotype helps prevent the accumulation of MTX over time in patients treated with HD-MTX. Moreover, in silico analysis has revealed that three of the 14 known MTX transporter genes, the SLC19A1, SLCO1A2, and SLC46A1 genes, are targets of miR-5189 and miR-595, while miR-6083 does not appear to target any of the transporters. In this way, specific genotypes of polymorphisms in these miRNAs regulate the transporter gene expression. For example, it was found that the mRNA levels of miR-595 increase when the TT genotype rs4909237 is present. That could reduce the expression of the three transport proteins, and consequently, both the biliary and urinary elimination of MTX would be decreased, potentially resulting in drug accumulation in plasma [3].

Other studies examined the relationship between serum MTX concentration and miRNA binding site polymorphisms in transporter genes. Wang et al. found that SLCO1A2 rs4149009 G > A polymorphism affects hsa-miR-324-3p and hsa-miR-1913 binding to SLCO1A2 mRNA and, in this way, affects the MTX transport process [89]. As a result, rs4149009 G > A polymorphism was linked with delayed MTX clearance in Chinese pediatric patients with ALL (rs4149009 GA or GG genotype). Another study conducted again in Chinese pediatric patients by the same team examined if there was a connection between serum MTX concentration levels and two different miRNA binding site polymorphisms in MTHFR (rs3737966 G > A and rs35134728 DEL/TTC). The results revealed that patients with the genotype rs3737001 A/A and rs3513001 TTC/TTC were significantly less likely to have a high concentration-to-dose (C/D) ratio of MTX compared to those without the genotype. Furthermore, the percentage of patients with high MTX concentration in their blood was substantially lower in those carriers of the genotype. These findings suggest that rs3737966 A or rs35134728 TTC could be functional variants with the potential to significantly influence serum MTX concentrations [90]. Likewise, Mishra et al. demonstrated that 829C→T polymorphism that is located near the miR-24 binding site in the 3′ UTR of the DHFR gene influences miR-24 function and, consequently, the expression of the DHFR gene. This can lead to an overexpression of DHFR and MTX resistance [91].

All these supporting results confirm that miRNAs could play a key role in the post-translational regulation of MTX-transporter genes and, in this way, affect the efficacy and toxicity of treatment.

**Table 2:** miRNA genetic variants and MTX toxicity.

miRNA	Genetic Variant	Toxicity	Effect on MTX Plasma Levels	References
miR-1208	rs264881	Hepatotoxicity	-	[86]
miR-1206	rs2114358	Oral mucositis Grade 3/4 leukopenia	-	[65,84]
miR-323b	rs56103835	Grade 3/4 anemia Gastrointestinal toxicities	Increase in MTX plasma levels	[65,87,92]
miR-4268	rs4674470	decreased risk of mucositis	-	[85]
miR-4751	rs8667	increased risk of diarrhea	-	[85]
miR-3117	rs12402181	decreased risk of vomiting	-	[85]
miR-200C	rs12904	74% decreased risk of hematological toxicity	-	[65]
miR-499A	rs3746444	77% decreased risk of hematological toxicity	-	[87]
miR-LET7A1	rs10739971	82% decreased risk of hematological toxicity	-	[87]
miR-149	rs2292832	Increased risk of neurological toxicity	-	[87]
miR-2053	rs10505168	Increased risk of neurological toxicity	-	[87]
miR-605	rs2043556	Increased risk of neurological toxicity and protection from infectious toxicity	-	[87]
miR-938	rs2505901	Protection from gastrointestinal and neurological toxicity	-	[87]
miR-5189	rs56292801	-	0.4-fold decrease in MTX plasma levels	[3]
miR-595	rs4909237	-	5.7-fold increase in MTX plasma levels	[3]
miR-6083	rs78790512	-	Prevention of MTX plasma accumulation	[3]

-: not studied.

## 7. Conclusions

MTX has been used for decades as the gold standard for the treatment of pediatric ALL. It is a fact that the administration of MTX alone or in combination with other drugs such as corticosteroids, vincristine, and L-asparaginase, has led to a significant improvement in the survival rate of children with ALL. Nevertheless, a great number of patients treated with MTX develop a variety of side effects like mucositis, nephrotoxicity, neurotoxicity, hepatotoxicity, bone marrow suppression, etc. These side effects of therapy can lead to serious complications and are also one of the main reasons for interruption or discontinuation of therapy, which may increase relapse risk. The pharmacogenetics of MTX have been studied extensively, and common SNPs in genes involved in the MTX cell pathway have been thought to influence the patient’s response to treatment. Among the genes that are well studied are the MTHFR gene, the SLCO1B1 gene, the TYMS gene, and the DHFR gene, as well as the SLC19A1 gene.

Recent research has demonstrated that miRNAs are involved in the transportation and metabolism of drugs, as well as the regulation of target gene expression. Consequently, it was proposed that miRNAs may be involved in the treatment response. To date, a number of studies have examined the relationship between miRNA polymorphism and MTX-induced toxicity in children with ALL. The challenge of modern medicine to predict which patient will develop toxicity in advance in order to adjust treatment from the beginning is very intriguing. It has also been demonstrated that alterations in the expression levels of a number of miRNAs play a role in both leukemogenesis and drug resistance; reversing these expression profiles could potentially enhance drug sensitivity and consequently lead to improved clinical results. However, miRNA involvement in the regulation of various pathways associated with MTX treatment is still largely unknown. Additional studies of MTX pharmacogenomics are needed, and confirmation of the results in larger prospective studies is required, as, currently, there is a great deal of contradictory evidence that has yet prevented definitive conclusions from being drawn. A pharmacogenomic panel of alleles relevant to all of the drugs used, especially in maintenance therapy, potentially including drugs such as vincristine and glucocorticoids, could also be useful prior to ALL therapy. Gene–gene interactions should be examined, and of course, the patient’s genotype should be considered in conjunction with other clinical parameters. The prospect of miRNA therapy and a more precise risk stratification based on miRNA profiles in ALL patients is very challenging as well. The results of this research are expected to be beneficial in order to elucidate the actual role of genetic variations in the pharmacologic control of ALL.

## Figures and Tables

**Table 1:** The role of miRNAs in prognosis ALL pediatric patients.

Prognosis of ALL	miRNA	Upregulation (↑) or Downregulation (↓)	References
Good prognosis and longer disease-free and overall survival	↑	miR-128b	[80]
	↑	miR-223	[80]
	↓	miR-16	[80]
Poor prognosis and response to therapy, increased occurrence of drug resistance, and increased risk of relapse	↑	miR-24	[80]
	↓	miR-335	[80]
	↓	miR-326	[80]
	↑	miR-155	[80]
	↓	miR-27a	[80]
	↓	miR-125b	[80]

## Data Availability

Data are contained within the article.
